# Secure OFDM with Peak-to-Average Power Ratio Reduction Using the Spectral Phase of Chaotic Signals

**DOI:** 10.3390/e23111380

**Published:** 2021-10-21

**Authors:** Mohamad F. Haroun, T. Aaron Gulliver

**Affiliations:** Department of Electrical and Computer Engineering, University of Victoria, P.O. Box 1700, STN CSC, Victoria, BC V8W 2Y2, Canada; mharoun@uvic.ca

**Keywords:** secure OFDM communication, chaotic phase sequence, PAPR reduction

## Abstract

In this paper, a new physical layer security technique is proposed for Orthogonal Frequency Division Multiplexing (OFDM) communication systems. The security is achieved by modifying the OFDM symbols using the phase information of chaos in the frequency spectrum. In addition, this scheme reduces the Peak to Average Power Ratio (PAPR), which is one of the major drawbacks of OFDM. The Selected Mapping (SLM) technique for PAPR reduction is employed to exploit the random characteristics of chaotic sequences. The reduction with this algorithm is shown to be similar to that of other SLM schemes, but it has lower computational complexity and side information does not have to be sent to the receiver. The security of this technique stems from the noise like behavior of chaotic sequences and their dependence on the initial conditions of the chaotic generator (which are used as the key). Even a slight difference in the initial conditions will result in a different phase sequence, which prevents an eavesdropper from recovering the transmitted OFDM symbols.

## 1. Introduction

The growth of wireless networks has led to a rapid increase in the transmission of information. This information varies greatly from data and video files to social media, e-trade, and personal and medical information. Further, the revolution in wireless network technologies driven by 5G and the Internet of Things (IoT) has resulted in a tremendous increase in the number and variety of mobile applications. These networks are characterized by dynamic topologies and limited device power. This makes security such as data authenticity, confidentiality and integrity an important issue. Cryptography is used in many systems and applications to provide security. However, this requires complex functions which consume time, power and resources. Further, cryptography can constrain the network topology and limit communications. An effective and efficient means of overcoming these obstacles is to take advantage of the physical properties of the wireless transmission medium to provide communication security, which is known as physical layer security [1].

Although noise, fading and interference are often considered to be undesirable features of wireless channels, they are useful from a security perspective. Many algorithms have been devised to limit the ability of an eavesdropper to gain information about confidential messages sent over a wireless channel. Further, the random nature of radio channels has been exploited to generate shared secret keys (key management) between legitimate users [2]. Orthogonal Frequency Division Multiplexing (OFDM) is widely used for data transmission in wireless communications because of its many advantages such as robustness to inter-symbol interference and frequency selective fading, and bandwidth efficiency. Further, chaos is a well-known phenomenon used in numerous security applications. In this paper, chaos is used to improve the performance of OFDM communications while providing security for the wireless link.

### 1.1. OFDM

Analog OFDM for multicarrier transmission was proposed in 1966 [3]. The popularity of OFDM is due to the fact that it overcomes the Inter-Symbol Interference (ISI) and Inter-Carrier Interference (ICI) problems common with wideband communication systems. This is achieved because the wideband frequency selective channel is divided into parallel frequency flat subchannels which simplifies the receiver design, particularly in terms of channel equalization. Digital baseband OFDM was proposed in [4] using an Inverse Fast Fourier Transform (IFFT) and a Fast Fourier Transform (FFT) for modulation and demodulation, respectively. This eliminates the subcarrier oscillators and coherent demodulators used in analog OFDM, which significantly reduces the cost and computational complexity.

One of the challenges with OFDM modulation is the potential for a high Peak to Average Power Ratio (PAPR). This has a negative effect on OFDM performance because the High Power Amplifier (HPA) is typically nonlinear due to efficiency and cost considerations. There are two solutions to this problem. The first is to use an expensive power amplifier with a large linear region (high dynamic range), but this lowers the power efficiency. The other solution is to reduce the PAPR by modifying the signal constellation before performing the IFFT at the transmitter.

The *PAPR* of an OFDM signal is defined as the ratio of the maximum instantaneous power to the average power

(1) $$\mathrm{PAPR}=\frac{\max_{0\leq n\leq NT}\left\{{\left|s(n)\right|}^{2}\right\}}{E\{{\left|s(n)\right|}^{2}\}},$$

where s(n) is the complex baseband signal in the time domain, *N* is the number of OFDM subcarriers, and *T* is the oversampling rate which is typically 4. The *PAPR* is also evaluated using the crest factor (*CF*) which is defined as

(2) $$\mathrm{CF}=\sqrt{\mathrm{PAPR}}.$$

The *PAPR* distribution can be expressed in terms of the Complementary Cumulative Distribution Function (*CCDF*) [5]

(3) CCDF=Prob{PAPR>z}.

With no *PAPR* reduction, the theoretical *CCDF* is given by

(4) $$\mathrm{CCDF}=1-{\left(1-e^{z^{2}}\right)}^{N},$$

where $z^{2}={\left|s\left(n\right)\right|}^{2}/\sigma^{2}$, and $\sigma^{2}$ is the variance of the complex samples s(n) which represents the average power of the baseband OFDM signals. This shows that as the number of subcarriers increases, the probability that the peak value exceeds a given value also increases, so the *PAPR* problem is exacerbated.

### 1.2. Chaos

Chaos is a natural phenomenon which has many interesting properties. One of the most important of these is sensitivity to initial conditions [6]. Chaos has been found to occur in many nonlinear dynamic systems. Although these systems are deterministic, the initial conditions cannot be predicted and the output never repeats.

Chaos has been used in communication systems since 1991 when Carroll and Pecora developed the first control technique to achieve synchronization between chaotic generators [7]. Chaotic signals are wideband, and as a consequence have been used as both carrier signals and spreading signals in Direct Sequence Spread Spectrum (DS-SS) systems. Chaos is an ideal candidate for use in cryptographic systems because it has desirable characteristics such as a broad spectrum, ergodicity, and high sensitivity to initial conditions, which are directly related to the basic cryptographic properties of confusion and diffusion [8]. Further, chaotic sequences have good randomness properties and a low cross-correlation. As a consequence, several chaos-based cryptosystems have been developed for wireless applications.

In this paper, chaos is used to develop a secure OFDM communication system. In particular, the phase of a chaotic sequence is used to modify the phase of the OFDM symbols. The randomness of the chaotic sequences is exploited to provide PAPR reduction using the Selected Mapping (SLM) technique. In particular, shifted versions of a chaotic phase sequence are combined with the OFDM symbol, and the sequence with the lowest PAPR is transmitted. A different chaotic sequence is used for each OFDM symbol.

As the chaotic phase can take any value from −π to π, quantization is used to reduce the complexity, but as will be shown in Section 3.3, this does not degrade the performance. It will be shown that the PAPR reduction achieved with this approach is similar to that with other PAPR reduction schemes. However, the proposed technique also provides secure communications and has low computational complexity. In addition, no side information need be sent to the receiver, which improves the bandwidth efficiency and security.

The security of the system depends on the initial conditions and the control parameters of the chaotic generator which are used as the encryption/decryption key. As the chaotic phase sequence is very sensitive to the initial conditions, even a slight difference in the key parameters will result in a completely different sequence, which prevents an eavesdropper from recovering the transmitted OFDM symbols.

The remainder of the paper is organized as follows. Section 2 provides an introduction to the SLM PAPR reduction technique. Section 3 presents the proposed algorithm including the generation of chaotic sequences, phase quantization, and reconstruction of the transmitted OFDM symbols at the receiver without side information. The performance is evaluated via simulation, and the PAPR reduction is compared with related techniques in the literature. The Symbol Error Rate (SER) is also presented to show the effect of not using side information. A security analysis is provided in Section 4, and finally some conclusions are given in Section 5.

## 2. Selected Mapping (SLM)

Most PAPR reduction techniques can be divided into two categories: distortion and distortionless. Distortion techniques such as clipping create in-band interference between subcarriers which affects the orthogonality and creates ISI, and out-of-band interference which produces ICI. Thus, additional processing is required to reduce these undesirable effects. Distortionless techniques such as selected mapping, coding, tone reservation, tone injection, and partial transmit sequence do not introduce ISI or ICI, but complexity can be an issue and side information typically has to be sent to the receiver, which reduces the overall data rate.

Selected Mapping (SLM) is a well-known distortionless technique. With SLM, the input data block is multiplied with *M* different phase sequences resulting in *M* OFDM symbols, and the symbol with the smallest PAPR is selected for transmission [9]. Therefore, the performance of this technique depends on the number of phase sequences and their design. Side information must be sent to inform the receiver which sequence was used, which requires $\left\lfloor \log_{2}M\right\rfloor$ bits. An algorithm to reduce the side information was proposed in [10], and an approach which does not require explicit side information was presented in [11], but the labels employed reduce the data rate. In [12,13], a comparison of phase sequence generation techniques was given. It was concluded that these sequences should be independent to maximize the PAPR reduction. In [14], a scheme based on exhaustive entropy and chaotic sequences was proposed. A chaotic attractor is used to generate *U* binary phase sequences, and exhaustive entropy is employed to select the *M* sequences for SLM. However, these techniques are very complex. Several approaches to reduce the complexity of SLM have been developed. In [15], conversion vectors obtained using the IFFT of the phase sequences were employed, while in [16], the IFFT structure was exploited to reduce the number of computations required.

## 3. The Proposed Algorithm

### 3.1. Chaotic Signals

Chaotic signals are random-like signals as their autocorrelation is similar to that of a random signal. These signals are unpredictable, and have a broad spectrum in the frequency domain. Because of these desirable properties, chaotic signals have been widely used in communication systems, particularly for security applications. Instead of the time domain representation typically used in the literature, the proposed algorithm uses the phase of the frequency components of the chaotic signal. The objective is to generate phase sequences which can be used to achieve both security and PAPR reduction, and thus these sequences should have random-like characteristics.

The logistic map is a very simple non-linear dynamical equation which exhibits chaotic behavior. It is considered here to generate the chaotic phase sequences, but other generators can be employed. This map is a one-dimensional (1D) discrete chaotic generator which has been used extensively in the literature because it can easily be implemented in hardware or software [17]. The dynamics of the logistic map are described by the difference equation

(5) $$x_{n+1}=rx_{n}\left(1-x_{n}\right),$$

where the state variable $x_{i}$ is a number between zero and one. The parameter *r* plays a critical role in the chaotic behavior, and it has been shown that for 3.56995≤r<4 the dynamics of the map exhibit chaotic behavior. The randomness of the state variable $x_{i}$ has been studied extensively in the literature [18]. Here, the initial value $x_{0}$ and the control parameter *r* are considered to be the secret key shared between legitimate users. Unlike other chaotic cryptosystems that have been developed for secure communications, the phase of the frequency spectrum of the chaotic sequence *x* is used in the proposed algorithm.

**Figure 1:**
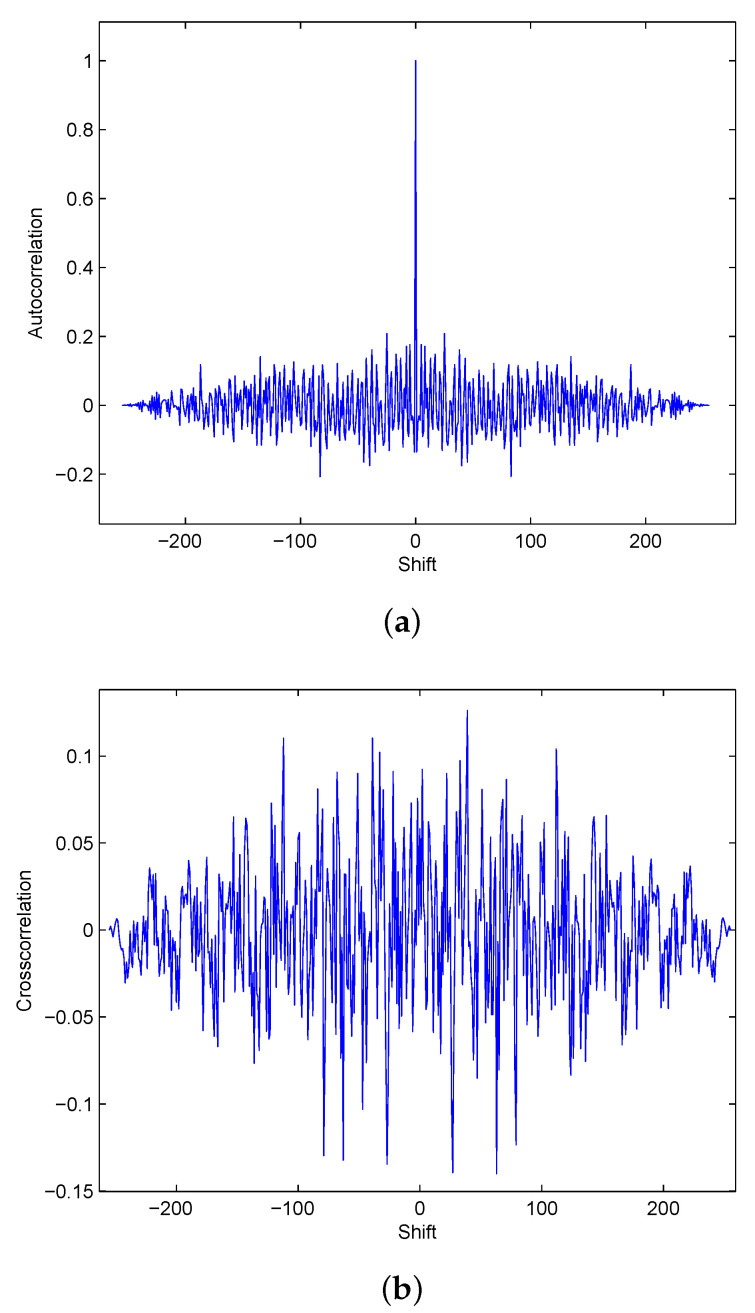
(**a**) The autocorrelation of the logistic map phase sequence for N=256, r=3.9 and $x_{0}=0.24$, and (**b**) the cross-correlation of this sequence with the corresponding phase sequence for r=3.9 and $x_{0}=0.37$.

The frequency spectrum of a chaotic signal has received little attention in the literature, but the phase of the frequency spectrum was examined in [19]. It was shown that the phase has a random distribution with a mean of zero over the main part of the frequency range. To test the randomness of chaotic phase sequences, the logistic map was used with r=3.9 and $x_{0}=0.24$ to generate a time domain sequence of length N=256. A 256 point DFT was used to obtain the corresponding phase sequence of the frequency spectrum. Figure 1a shows the autocorrelation of this sequence, *x*, and Figure 1b shows the cross-correlation of *x* and a second sequence *y* generated using r=3.9 and $x_{0}=0.37$. These results indicate that the chaotic phase sequences have random characteristics. The correlation coefficient for sequences *x* and *y* is given by

(6) $$r_{xy}=\frac{C_{xy}}{\sigma_{x}\sigma_{y}},$$

where $\sigma_{x}$ and $\sigma_{y}$ are the corresponding variances and $C_{xy}$ is the autocovariance given by

$$C_{xy}=E\left[\left\{X-\mu_{x}\right\}\left\{Y-\mu_{y}\right\}\right],$$

with means $\mu_{x}$ and $\mu_{y}$. The correlation coefficient for the phase sequences *x* and *y* obtained using the parameters above is 0.0261, which shows that there is minimal correlation between them [20]. Thus, it can be concluded that the phase of a chaotic signal is a good candidate for use in secure communications applications. As these sequences have random characteristics, it is expected that they can also be used to improve the PAPR.

### 3.2. Chaotic SLM

As stated in the previous section, with the SLM technique *M* different sequences are created from the data to be transmitted, and the one with the smallest PAPR is selected for transmission. The generation of these sequences increases the computational complexity at the transmitter. The proposed algorithm benefits from the random characteristics of chaotic phase sequences to produce the *M* sequences with low complexity. The chaotic generator is used to obtain a phase sequence of length *N*, and using cyclic shifts, *M* versions of this sequence are obtained. The length of the cyclic shift *L* is given by

**Figure 2:**
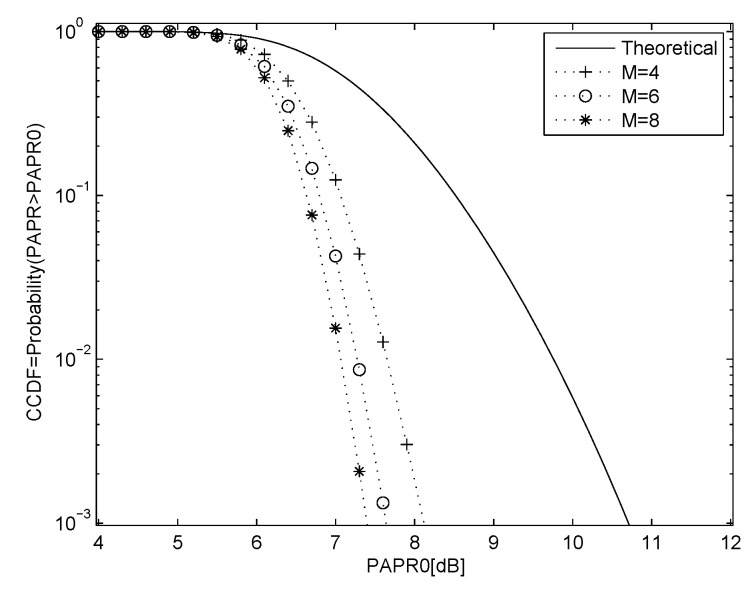
PAPR performance for 16-QAM modulation with N=128 and M=4,6 and 8 chaotic SLM sequences, and the theoretical result with no PAPR reduction.

**Figure 3:**
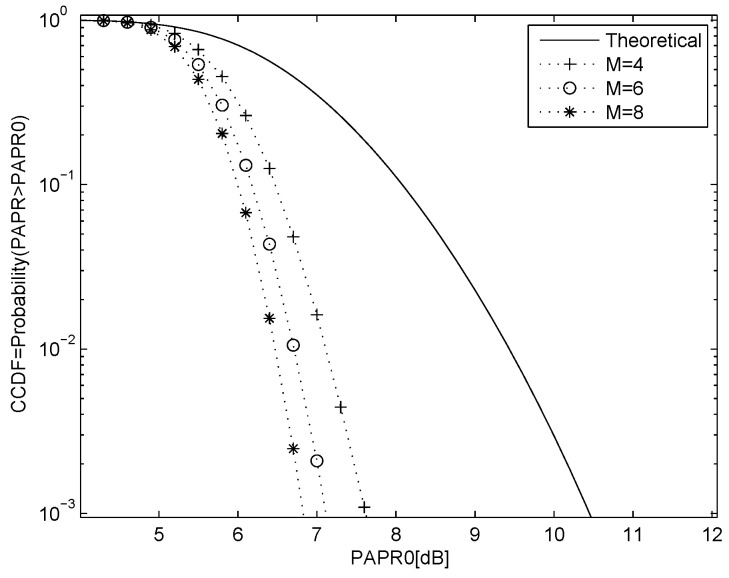
PAPR performance for QPSK modulation with N=64 and M=4,6 and 8 chaotic SLM sequences, and the theoretical result with no PAPR reduction.

(7) L=⌊N/M⌋,

where ⌊z⌋ denotes the largest integer less than or equal to *z*. The *M* sequences are used to modify the phase of the OFDM data, and the sequence with the smallest PAPR is selected for transmission. Figure 2 shows the PAPR performance using 16-QAM modulation with N=128 and M=4, 6 and 8, and the theoretical performance with no PAPR reduction. Figure 3 shows the PAPR performance for QPSK with N=64 and M=4, 6 and 8, and the theoretical performance with no PAPR reduction.

### 3.3. Quantization of the Chaotic Phase Sequences

**Figure 4:**
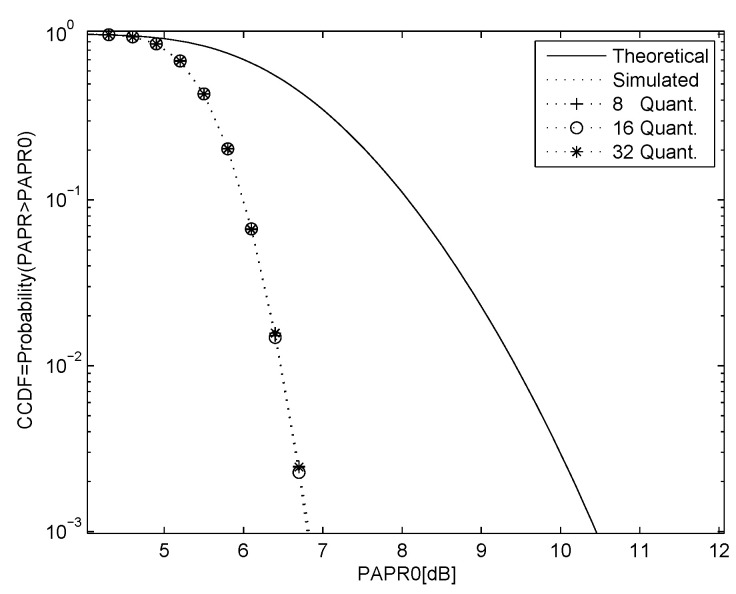
PAPR performance for QPSK modulation with N=64 and 8 chaotic SLM sequences with K=8,16 and 32 phase regions, and the theoretical result with no PAPR reduction.

As the phase of the chaotic signal is continuous, the complexity of combining the corresponding phase sequences with the OFDM symbols is high. To reduce this complexity, the phase space is partitioned into a number of regions and the phase in each region is assigned a fixed value. Then, a lookup table can be used instead of multiplications. For example, if the OFDM data constellation has *S* points and the number of phase regions is *K*, the look-up table will have size S×K. Figure 4 shows the PAPR performance with QPSK modulation, N=64 and M=8 chaotic SLM sequences using K=8,16 and 32 phase quantization regions. Comparing this figure with the corresponding results in Figure 3 indicates that the quantization has a minimal effect on the performance.

The number of regions *K* has an impact on the security of the algorithm, as a greater number of regions increases the complexity, making it harder for an attacker to determine the OFDM symbols. However, this also increases the size of the look-up table. As the PAPR performance is not greatly affected by the value of *K*, it can be chosen based on the available computational resources and required security.

**Figure 5:**
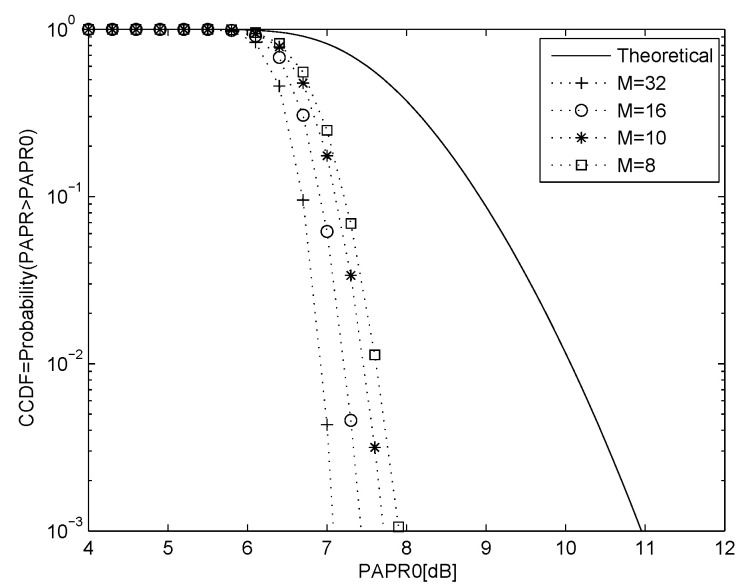
PAPR performance of the proposed chaotic SLM compared with SLM techniques in the literature for 16-QAM with N=256 and M=8, 10, 16 and 32 sequences, and the theoretical result with no PAPR reduction.

The proposed system is now compared with several SLM techniques in the literature. Figure 5 presents the PAPR performance of 16-QAM with N=256 and M=8,10,16 and 32. For M=32 the proposed algorithm is slightly better than the approach in [21], and for M=16 is similar to the results in [22]. Compared with the technique in [23], with M=10 the proposed solution is 0.7 dB better at $\mathrm{CCDF}=10^{-3}$. For M=8, it is better than the results in [24], and is much better than those in [25]. Note that the proposed algorithm is superior from the security perspective, and has low computational complexity.

### 3.4. Data Recovery without Side Information

With most SLM PAPR reduction techniques, the original data are recovered at the receiver using side information sent from the transmitter to identify the sequence that was used. However, the phase sequence employed can be predicted at the receiver using the proposed algorithm without side information. This is based on the fact that the transmitter and receiver have the same phase sequences from the chaotic generator. The Euclidean distance between the recovered symbols $q_{i}^{j}$ using the *j*th shifted phase sequence and the OFDM data constellation $p_{i}$ is

(8) $$d\left(j\right)=\sqrt{\sum_{i=1}^{N}{\left(p_{i}-q_{i}^{j}\right)}^{2}},j=1,\ldots ,M.$$

These distances are calculated at the receiver to predict the chosen phase sequence, as described below.

At the receiver, the same chaotic phase sequence as at the transmitter is generated based on the shared key between them, so it has the same *M* shifted sequences. The received signal after the IFFT is combined with these sequences to remove the effect of the phase sequences. Then the Euclidean distance (8) is calculated for the resulting sequences, and the one corresponding to the lowest value of d(j) is considered as the original data. This approach requires that the chaotic phase constellation differ from the constellation of the data modulation, which is typically PSK or QAM. Thus, the transmitted OFDM symbol has a constellation which differs from the data constellation. At the receiver, if the received OFDM symbol is modified with the shifted phase sequence used by the transmitter, the resulting constellation will be the data constellation. However, if an incorrect phase sequence is used at the receiver, the resulting sequence will still be a combination of the phase sequence and data constellations. Thus, the correct phase sequence should result in the lowest Euclidean distance. Note that the chaotic phase constellation points have unit magnitude, so that although the transmitted symbols are from a new constellation, the magnitudes are unchanged, so the proposed algorithm does not increase the average transmit power as with other PAPR reduction approaches.

### 3.5. Algorithm Summary

The proposed algorithm can be summarized as follows.

- Using the initial conditions, a chaotic generator is employed to create a chaotic sequence in the time domain. The length of this chaotic sequence is equal to the length of the OFDM symbol. Subsequent chaotic sequences are obtained from the chaotic generator using the state from the previous sequence.
- A Discrete Fourier Transform (DFT) is used to obtain the phase information from the frequency spectrum of the sequence.
- The phase information is quantized to reduce the implementation complexity and increase the security of the system. The quantized phase sequence is used to change the phase of the OFDM symbol.
- At the receiver, only a legitimate user is able to generate the phase sequence and recover the original OFDM symbol.

### 3.6. Performance Results

In this section, an OFDM system with N=128 is considered with QPSK and 16-QAM modulation. The number of SLM sequences is M=8, and these are obtained using the logistic map as the chaotic generator. The initial conditions and control parameter are identical at the transmitter and receiver (the legitimate users), and are $x_{0}=0.24$ and r=3.9.

**Figure 6:**
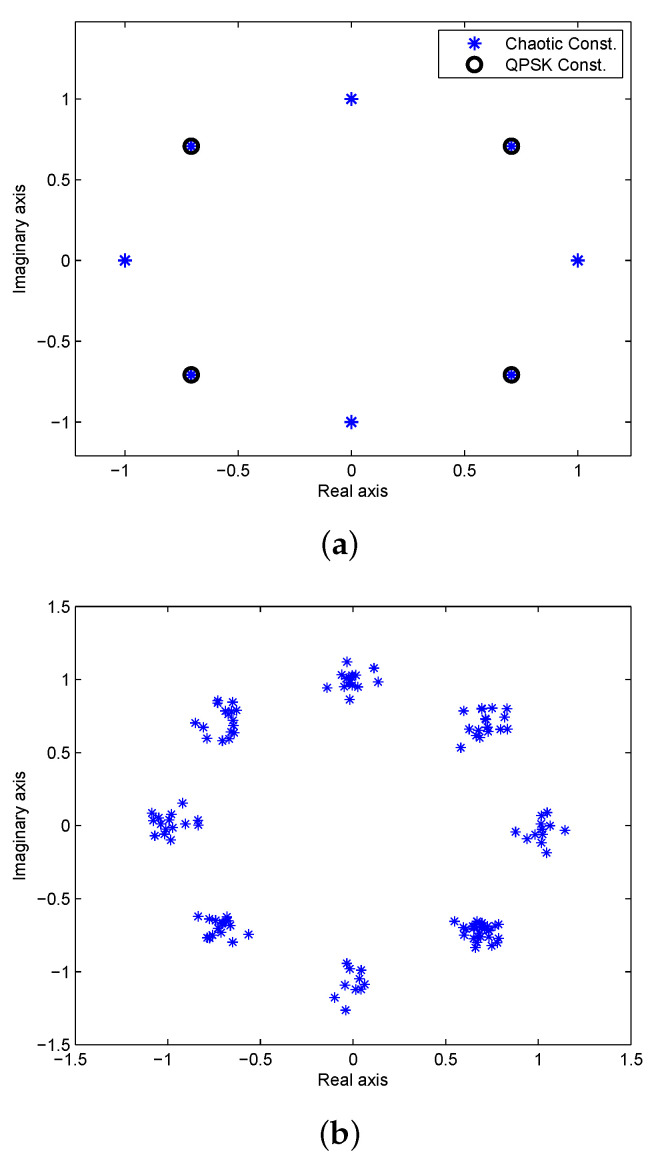
(**a**) The constellations of the quantized chaotic phase sequences (K=8) and QPSK, and (**b**) the received OFDM symbol constellation for SNR =20 dB.

**Figure 7:**
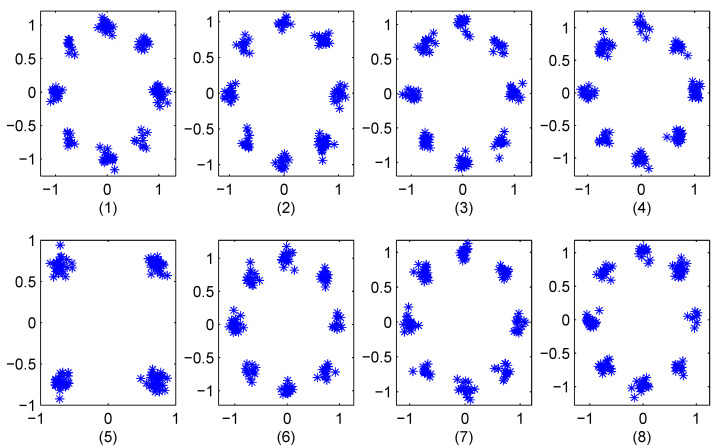
The constellations for the 8 recovered OFDM symbols with QPSK modulation for SNR =20 dB.

**Figure 8:**
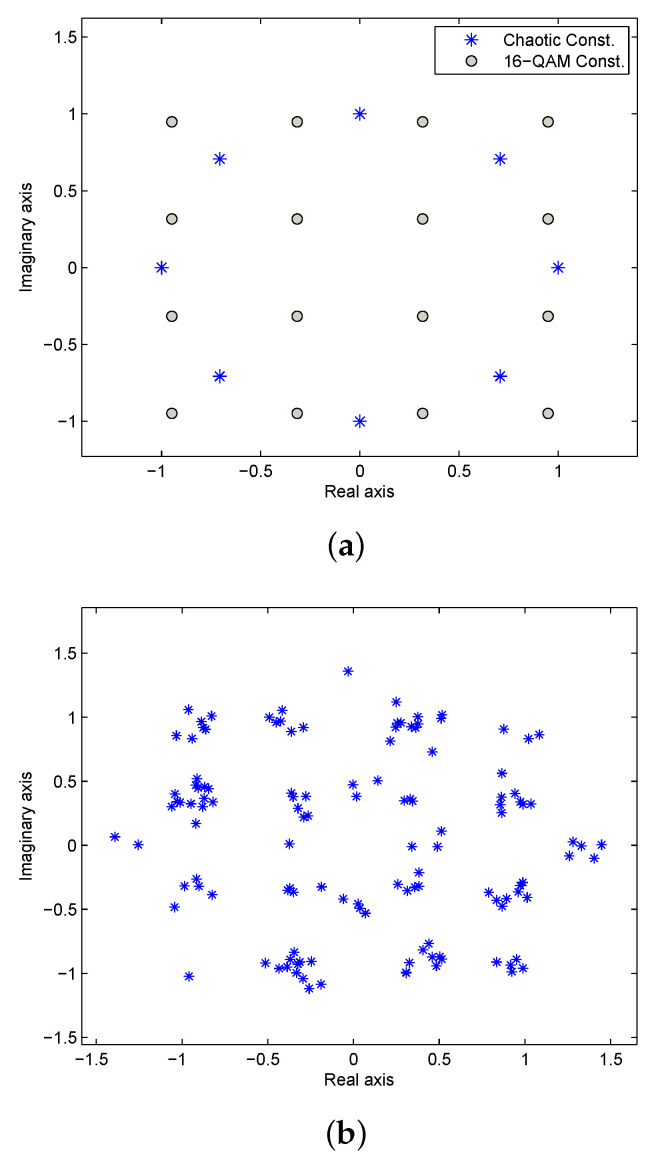
(**a**) The constellations of the quantized chaotic phase sequences (K=8) and 16-QAM, and (**b**) the received OFDM symbol constellation for SNR =20 dB.

**Figure 9:**
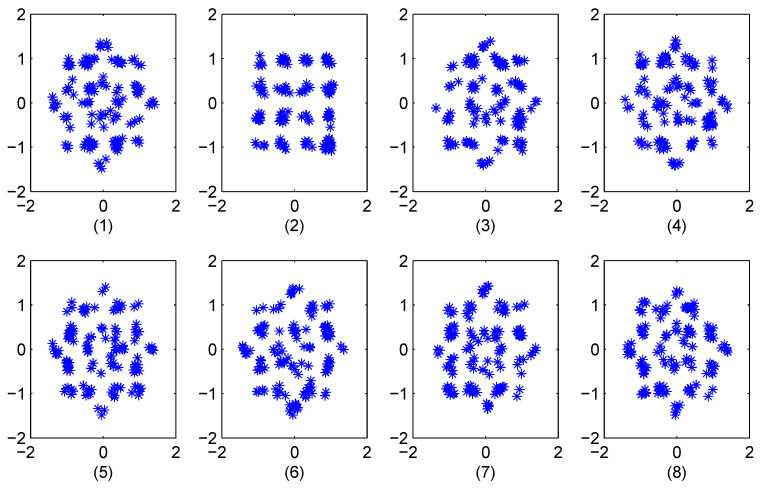
The constellations of the 8 recovered OFDM symbols with 16-QAM modulation for SNR =20 dB.

We first consider an Additive White Gaussian Noise (AWGN) channel with SNR =20 dB and K=8 phase sequence constellation points. Figure 6a presents the quantized chaotic phase sequence and QPSK constellations, and Figure 6b the constellation of the corresponding received OFDM symbol. The results of reconstructing the OFDM data using the 8 phase sequences are given in Figure 7. This shows that chaotic phase sequence 5 is the most likely one employed at the transmitter. The corresponding Euclidean distance calculations are given in the first row of Table 1. As expected, the Euclidean distance for sequence 5 is the lowest, so the receiver selects this sequence to recover the data (which is correct). Figure 8 presents the constellations for 16-QAM with K=8, and Figure 9 shows the results of removing the effects of the phase sequences on the received OFDM symbol. In this case, sequence 2 appears to be correct (which is indeed the case), and this is confirmed by the Euclidean distances in the second row of Table 1.

**Figure 10:**
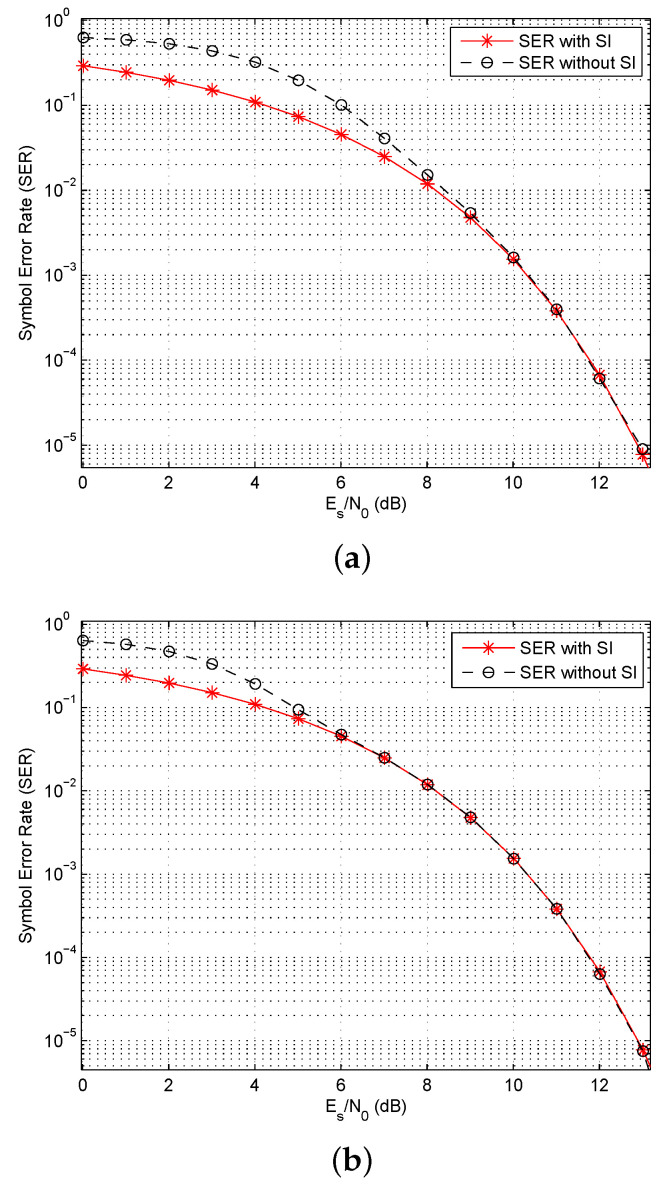
The Symbol Error Rate (SER) with QPSK modulation using length (**a**) N=32, and (**b**) N=64 OFDM symbols with and without Side Information (SI) over an AWGN channel.

**Figure 11:**
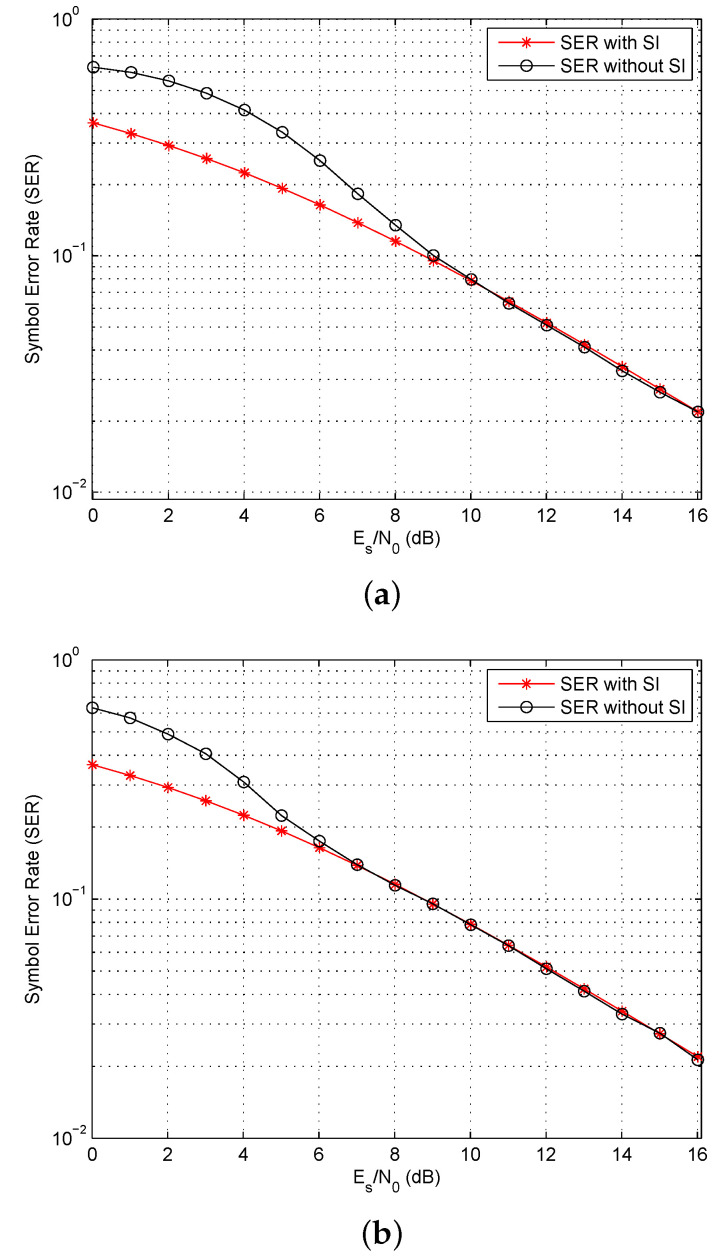
The Symbol Error Rate (SER) with QPSK modulation using length (**a**) N=32, and (**b**) N=64 OFDM symbols with and without Side Information (SI) over a Rayleigh fading channel.

As the proposed algorithm makes a decision based on the Euclidean distance without using side information, the SER performance will be affected. Thus, the SER was obtained for QPSK modulation with and without side information (but without considering the loss in data rate in the former case). Figure 10 presents the results for N=32 and 64 over an AWGN channel. This shows that the performance is identical for high SNRs. Further, increasing the length of the OFDM symbols improves the performance, as the difference is insignificant for an SNR of 6 dB with N=64, but for N=32 the SNR should be greater than 9 dB. Given that in practical OFDM systems *N* is typically 256 or larger, the loss in performance due to not using side information is negligible. Further, this eliminates the loss in data rate due to transmitting this information. The corresponding performance over a Rayleigh fading channel is shown in Figure 11. This indicates that the fading channel performance is very similar to that in an AWGN channel in terms of the SNR values where the SER with side information is the same as the SER without side information. Thus fading does not affect the reliability of the proposed algorithm.

## 4. Security Analysis

In the literature, the outputs of chaotic generators in the time domain are used to obtain random sequences of zeros and ones. These random sequences are used to encrypt the data either directly via modulo 2 addition, or indirectly as a key for a traditional cryptographic algorithm. The security depends on the control parameters and initial values of the chaotic generator, which constitute the shared key between legitimate users. The complexity of the chaotic system makes it hard for an eavesdropper to predict the key. However, some cryptanalysis methods exploit the synchronization of the chaotic generator with the transmitted signal, and some systems can be broken even when the control parameters and initial values are not known exactly.

**Figure 12:**
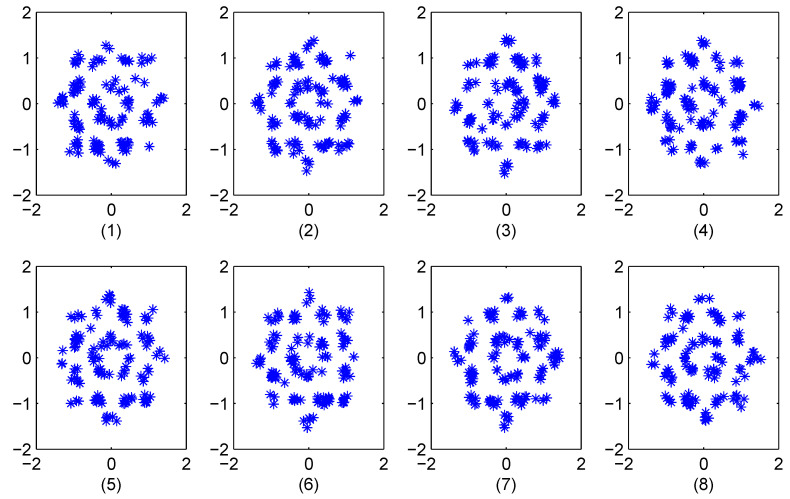
The 16-QAM constellations obtained by an eavesdropper with initial conditions different than at the transmitter.

Conversely, the proposed algorithm uses the phase of the frequency response of the chaotic signal. As shown in Section 3.1, the phase sequences of two different chaotic signals are uncorrelated, so the phase sequences for two parties having different keys will also be uncorrelated. Consider the transmitted 16-QAM OFDM symbol in Section 3.6 and an eavesdropper who has initial values r=3.9 and $x_{0}=0.67$. Figure 12 shows the constellations of the recovered 16-QAM OFDM symbols with K=8 at the eavesdropper. The similarity between these constellations makes it impossible to predict the transmitted OFDM symbol. This is confirmed by the corresponding Euclidean distances in Table 2, which are very close.

Note that quantizing the chaotic phase sequences prevents an eavesdropper from predicting the actual phases. Further, transmitting side information as is common with PAPR reduction techniques in the literature can compromise the security of the system, but the proposed approach does not require that this information be sent. This prevents an eavesdropper from obtaining information which can assist in predicting the OFDM data. Finally, the non-periodicity of the chaotic signal guarantees that the phase sequences differ for each OFDM symbol.

The control parameters and the initial values of the chaotic generator form the key shared between legitimate users. The key length is a function of the dimensionality of the chaotic generator, the number of control parameters, and the precision of the hardware or software in use. As the chaotic generator is described by difference equations and each equation has an initial value, higher dimensional generators will have a longer key length. Chaotic systems are sensitive to very small signal deviations, so that once the sensitivity threshold of the hardware or the software is reached, the orbit of the chaotic system will be affected. In [26], it was shown that a Lorenz generator implemented using double precision floating point has a key space of $10^{39}$. This key length is greater than $2^{100}$ and so provides significant robustness against a brute-force attack [27]. On the other hand, using a high-dimensional chaotic generator means more computations per key bit. As a result, the chaotic generator should be chosen according to the required level of security and the available computational resources. In this paper, the logistic map is used for illustration purposes.

## 5. Conclusions

In this paper, a secure OFDM communication system has been proposed. The security is based on the randomness of chaotic phase sequences which are combined with the OFDM symbols. Further, the Selected Mapping (SLM) technique was employed with these sequences to achieve a reduction in the PAPR. A single chaotic sequence was used to generate all of the SLM sequences for a given symbol using cyclic shifts so that the complexity is low compared to other techniques in the literature. It was shown that quantizing the chaotic phase sequence has a minimal effect on the performance, but significantly reduces the computational complexity. The non-periodicity of the chaotic signal guarantees a unique phase sequence for every OFDM symbol. The key length is determined by the chaotic generator and the hardware or software used. A higher-dimensional generator will have a longer key length. In addition, a technique was presented to eliminate the need to send side information to the receiver to recover the transmitted data, which improves the bandwidth efficiency and security compared to other approaches. Results were presented which show that the proposed technique has PAPR performance which is comparable to that with other solutions, but it also provides secure communications.

## Figures and Tables

**Table 1:** Euclidean distances for the M=8 SLM chaotic phase sequences.

Sequence No.	1	2	3	4	5	6	7	8
QPSK	6.66	5.67	6.02	5.87	1.10	5.77	5.90	5.66
16-QAM	2.68	1.13	2.43	2.72	2.57	2.92	2.54	2.70

**Table 2:** The Euclidean distances for the M=8 SLM sequences at an eavesdropper.

Sequence No.	1	2	3	4	5	6	7	8
16-QAM	2.48	2.55	2.64	2.70	2.44	2.44	2.73	2.54

## Data Availability

There is no data associated with this research.
